# Competitive endogenous RNA networks: integrated analysis of non-coding RNA and mRNA expression profiles in infantile hemangioma

**DOI:** 10.18632/oncotarget.23946

**Published:** 2018-01-04

**Authors:** Jun Li, Qian Li, Ling Chen, Yanli Gao, Bei Zhou, Jingyun Li

**Affiliations:** ^1^ Department of Plastic and Cosmetic Surgery, Maternal and Child Health Medical Institute, The Affiliated Obstetrics and Gynecology Hospital of Nanjing Medical University, Nanjing Maternity and Child Health Care Hospital, Nanjing 210004, China

**Keywords:** non-coding RNA, lncRNA, miRNA, mRNA, infantile hemangioma

## Abstract

Infantile hemangioma (IH) is the most common vascular tumour in infants. The pathogenesis of IH is complex and poorly understood. Therefore, achieving a deeper understanding of IH pathogenesis is of great importance. Here, we used the Ribo-Zero RNA-Seq and HiSeq methods to examine the global expression profiles of protein-coding transcripts and non-coding RNAs, including miRNAs and lncRNAs, in IH and matched normal skin controls. Bioinformatics assessments including gene ontology (GO) and kyoto encyclopedia of genes and genomes (KEGG) pathway analyses were performed. Of the 16370 identified coding transcripts, only 144 were differentially expressed (fold change ≥ 2, *P* ≤ 0.05), including 84 up-regulated and 60 down-regulated transcripts in the IH samples compared with the matched normal skin controls. Gene ontology analysis of these differentially expressed transcripts revealed 60 genes involved in immune system processes, 62 genes involved in extracellular region regulation, and 35 genes involved in carbohydrate derivative binding. In addition, 256 lncRNAs and 142 miRNAs were found to be differentially expressed. Of these, 177 lncRNAs and 42 miRNAs were up-regulated in IH, whereas 79 lncRNAs and 100 miRNAs were down-regulated. By analysing the Ribo-Zero RNA-Seq data in combination with the matched miRNA profiles, we identified 1256 sponge modulators that participate in 87 miRNA-mediated, 70 lncRNA-mediated and 58 mRNA-mediated interactions. In conclusion, our study uncovered a competitive endogenous RNA (ceRNA) network that could further the understanding of the mechanisms underlying IH development and supply new targets for investigation.

## INTRODUCTION

Infantile hemangioma (IH) is the most common vascular tumour in childhood, affecting 4% to 5% of infants worldwide [1]. Hemangiomas can show severe progression, which leads to tissue and organ damage that in some cases becomes life-threatening. Clinical treatment varies, including steroids, interferon-alfa, and β-blocker propranolol [2, 3]. However, no definitive therapy is available for IH due to the adverse effects of each drug. The risk factors for IH include preterm birth and placental anomalies [4]. In most cases, IH has a unique clinical course with proliferation and involution phases [5]. Numerous genes involved in IH have been identified. However, the pathogenesis and cause of hemangioma remain largely unknown.

The competitive endogenous RNA (ceRNA) hypothesis proposes that RNA transcripts, both coding and non-coding, compete for post-transcriptional control and coregulate each other using microRNA response elements (MREs) [6, 7]. Mounting evidence has shown that long non-coding RNAs and messenger RNAs can function as ceRNAs in diverse physiological and pathophysiological states such as myogenesis, melanoma development and cancer [8–11]. A recent study profiled the expression of distinct long non-coding RNAs (lncRNAs) in infantile hemangioma using microarray analysis and suggested that lncRNAs regulated several genes with important roles in angiogenesis [12]. Endothelial and circulating C19MC microRNAs are biomarkers of infantile hemangioma [13]. Additionally, integrative meta-analysis identified microRNA-regulated networks in infantile hemangioma [14]. However, the role of the ceRNA network in IH has not been elucidated.

In this study, we used Ribo-Zero RNA-Seq and HiSeq to examine the global expression profiles of protein-coding transcripts and non-coding RNAs, including miRNAs and lncRNAs, in IH and matched normal skin controls. Subsequently, gene ontology and pathway analysis displayed that, compared with the matched normal skin controls, many processes over-represented in IH were related to immune system processes, extracellular region regulation, and carbohydrate derivative binding. Further ceRNA network analysis identified 1256 sponge modulators including 87 miRNA-mediated, 70 lncRNA-mediated and 58 mRNA-mediated interactions. Our study may help expand understanding of the roles of the transcriptome, particularly non-coding transcripts, in the mechanisms underlying IH development and provide new research directions.

## RESULTS

### Differential expression profiles and bioinformatics analysis of mRNAs in IH compared with matched normal skin controls

**Figure 1:**
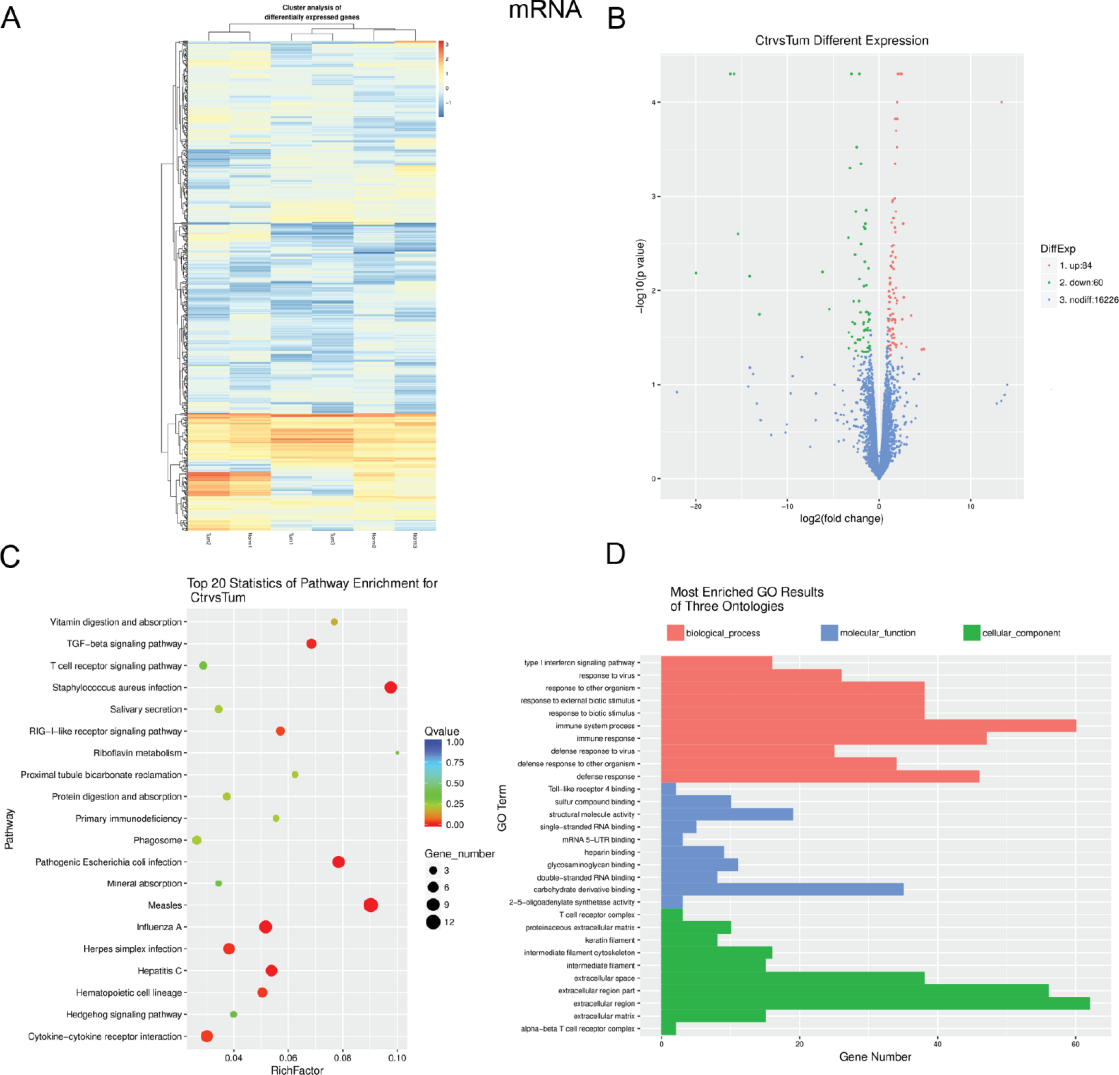
Expression profiles, Gene ontology (GO) terms and pathways for differentially expressed mRNAs between infantile hemangioma and adjacent normal skin tissues (**A**) Hierarchical clustering shows a distinguishable mRNA expression profiling among groups. (**B**) Volcano analysis exhibit differentially expressed mRNAs. Red dots represent up-regulated genes. Green dots illustrate down-regulated genes. (**C**) The top 20 pathways that are associated with the coding genes are listed. The enrichment *Q* value or false discovery rate correct the *p* value for multiple comparisons. *P* values are calculated using Fisher’s exact test. The term/pathway on the vertical axis is drawn according to the first letter of the pathway in descending order. The horizontal axis represents the enrichment factor, i.e., (the number of dysregulated genes in a pathway/the total number of dysregulated gene)/(the number of genes in a pathway in the database/the total number of genes in the database). Top 20 enriched pathways are selected according to the enrichment factor value. The selection standards were the number of genes in a pathway ≥4. The different colours from green to red represent the *Q* value (False discovery rate value). The different sizes of the round shapes represent the gene count number in a pathway. (**D**) Most enriched GO terms of the three ontologies that are associated with the differentially expressed coding genes are listed. The horizontal axis represents the gene number. The term/GO on the vertical axis is drawn according to the first letter of the GO in descending order. Red bar represents the biological process, blue bar displays the molecular function, and green bar illustrates the cellular component. Norm or Ctr, matched normal skin tissue; Tum, infantile hemangioma skin tissue.

To profile differentially expressed mRNAs, lncRNAs and miRNAs in IH, we performed RNA-seq on 3 IH samples and matched normal skin controls. We used an Illumina HiseqXTen platform (Illumina, San Diego, CA) for sequencing with (2 × 150 bp) the paired-end module. Fold changes (IH vs. matched normal skin controls) and *p* values were calculated from the normalized expression levels. Hierarchical clustering showed distinguishable mRNA expression patterns among the samples (Figure 1A). Up to 144 mRNAs were differentially expressed in the IH samples compared with the matched normal skin controls (fold change ≥2, *P* ≤ 0.05; for a list of differentially expressed mRNAs, see Table 1). A total of 84 and 60 mRNAs were up-regulated or down-regulated, respectively, by more than two-fold in IH vs. adjacent normal skin tissues (*P* < 0.05) (Figure 1B). KEGG Pathway analysis indicated that the chemokine, NF-kappa B and TGF-beta signalling pathways, as well as cell adhesion molecules (CAMs), were mostly found in the IH samples compared with matched normal skin (Figure 1C). In addition, gene ontology (GO) analysis revealed that numerous biological processes, molecular functions and cellular components were involved. Many of the processes that are deregulated in IH were related to immune system processes, carbohydrate derivative binding and extracellular region regulation (Figure 1D).

**Table 1:** List of up-regulated and down-regulated mRNAs detected using RNA-seq (FC ≥ 2.83, *P* < 0.05)

Gene Name	log2(Tum/Ctr)	up-or-down	*P*_value	Description
MPO	–6.18461	down	0.00635	myeloperoxidase
MAGEB2	–5.44166	down	0.0158	MAGE family member B2
CD8A	–3.35204	down	0.00275	CD8a molecule
BPI	–3.31652	down	0.0279	bactericidal/permeability-increasing protein
PGLYRP1	–3.3111	down	0.0412	peptidoglycan recognition protein 1
LOC283788	–3.17651	down	0.0005	FSHD region gene 1 pseudogene
IL18R1	–3.02589	down	5.00E-05	interleukin 18 receptor 1
ADCYAP1	–2.96929	down	0.031	adenylate cyclase activating polypeptide 1
CXCL13	–2.90547	down	0.02165	C-X-C motif chemokine ligand 13
MS4A1	–2.78553	down	0.013	membrane spanning 4-domains A1
SERPINB4	–2.67117	down	0.02235	serpin family B member 4
MMP12	–2.65413	down	0.02265	matrix metallopeptidase 12
**PIP**	**–2.63046**	**down**	**0.00195**	**prolactin induced protein**
LEFTY2	–2.59114	down	0.0361	left-right determination factor 2
IL13RA2	–2.54593	down	0.04385	interleukin 13 receptor subunit alpha 2
CSMD3	–2.54162	down	0.00145	CUB and Sushi multiple domains 3
OR51E1	–2.45263	down	0.0003	olfactory receptor family 51 subfamily E member 1
PTH2R	–2.32814	down	0.03325	parathyroid hormone 2 receptor
ADRB3	–2.30562	down	0.0493	adrenoceptor beta 3
CCL4L2	–2.18575	down	0.01295	C-C motif chemokine ligand 4 like 2
ERAP2	–2.14489	down	5.00E-05	endoplasmic reticulum aminopeptidase 2
LTF	–2.14431	down	0.00755	lactotransferrin
PADI4	–2.08682	down	0.0333	peptidyl arginine deiminase 4
OR51E2	–2.0297	down	0.01705	olfactory receptor family 51 subfamily E member 2
FKBP5	–1.98625	down	0.00045	FK506 binding protein 5
FOLH1	–1.97677	down	0.0032	folate hydrolase (prostate-specific membrane antigen) 1
CD3G	–1.862	down	0.04425	CD3g molecule
COL6A5	–1.72947	down	0.0316	collagen type VI alpha 5
TUBBP5	–1.70945	down	0.0228	tubulin beta pseudogene 5
CLEC4M	–1.6691	down	0.0217	C-type lectin domain family 4 member M
S100A9	–1.66717	down	0.0021	S100 calcium binding protein A9
DIO3	–1.65761	down	0.009	deiodinase, iodothyronine, type III
LOC645752	–1.64348	down	0.0449	golgin A6 family member A pseudogene
CD3E	–1.61303	down	0.0266	CD3e molecule
TNNT3	–1.55491	down	0.00495	troponin T3, fast skeletal type
S100A8	–1.53696	down	0.0022	S100 calcium binding protein A8
FUT9	–1.50479	down	0.00195	fucosyltransferase 9
KRT31	1.50307	up	0.02565	keratin 31
KRTAP11-1	1.51562	up	0.00975	keratin associated protein 11-1
KRT85	1.51808	up	0.01765	keratin 85
KRT81	1.54671	up	0.0292	keratin 81
KRT34	1.54769	up	0.03615	keratin 34
EPSTI1	1.574	up	0.0017	epithelial stromal interaction 1 (breast)
IFI6	1.57562	up	0.0033	interferon alpha inducible protein 6
IFI35	1.59026	up	0.0059	interferon induced protein 35
KRTAP3-2	1.61219	up	0.0107	keratin associated protein 3-2
KC6	1.62074	up	0.0318	keratoconus gene 6
KRT83	1.65762	up	0.0203	keratin 83
OAS3	1.69667	up	0.00105	2'-5'-oligoadenylate synthetase 3
USP18	1.72046	up	0.00215	ubiquitin specific peptidase 18
CYP26B1	1.74162	up	0.00045	cytochrome P450 family 26 subfamily B member 1
LNX1-AS2	1.74788	up	0.022	LNX1 antisense RNA 2
IFI44	1.75064	up	0.00015	interferon induced protein 44
TNFRSF4	1.75918	up	0.0375	tumor necrosis factor receptor superfamily member 4
LOC339975	1.76055	up	0.02065	uncharacterized LOC339975
CLDN11	1.7733	up	0.012	claudin 11
KRT35	1.78149	up	0.0024	keratin 35
KRT33A	1.80012	up	0.00445	keratin 33A
OAS2	1.83729	up	0.00145	2'-5'-oligoadenylate synthetase 2
KRT86	1.85155	up	0.0094	keratin 86
DCD	1.85338	up	0.0401	dermcidin
ACAN	1.85416	up	0.0002	aggrecan
PKD1L2	1.90338	up	0.0378	polycystin 1 like 2 (gene/pseudogene)
SCGB1B2P	1.91013	up	0.01375	secretoglobin family 1B member 2, pseudogene
CMPK2	1.92066	up	0.00015	cytidine/uridine monophosphate kinase 2
ADAMTS18	1.95193	up	0.0003	ADAM metallopeptidase with thrombospondin type 1 motif 18
KRT33B	1.9594	up	0.0128	keratin 33B
IFIT1	1.97285	up	0.0001	interferon induced protein with tetratricopeptide repeats 1
MX1	1.99777	up	0.00015	MX dynamin like GTPase 1
RSAD2	2.07171	up	5.00E-05	radical S-adenosyl methionine domain containing 2
**IFI44L**	**2.31465**	**up**	**5.00E-05**	**interferon induced protein 44 like**
LINC00487	2.42803	up	0.0367	long intergenic non-protein coding RNA 487
**ISG15**	**2.47094**	**up**	**5.00E-05**	**ISG15 ubiquitin-like modifier**
SULT1A2	2.52975	up	0.02025	sulfotransferase family 1A member 2
DMC1	2.61725	down	0.00415	DNA meiotic recombinase 1
FAM132B	2.69101	up	0.0118	-
NRIR	2.97361	up	0.0399	negative regulator of interferon response (non-protein coding)
MTHFD2P1	3.49824	up	0.01845	methylenetetrahydrofolate dehydrogenase (NADP+ dependent) 2, methenyltetrahydrofolate cyclohydrolase pseudogene 1
OR8B2	4.67189	up	0.0423	olfactory receptor family 8 subfamily B member 2
LOC101929128	4.88337	up	0.0419	uncharacterized LOC101929128

Ctr, matched normal skin tissue; Tum, infantile hemangioma skin tissue.

### Bioinformatics analysis of lncRNAs in IH compared with matched normal skin controls

**Figure 2:**
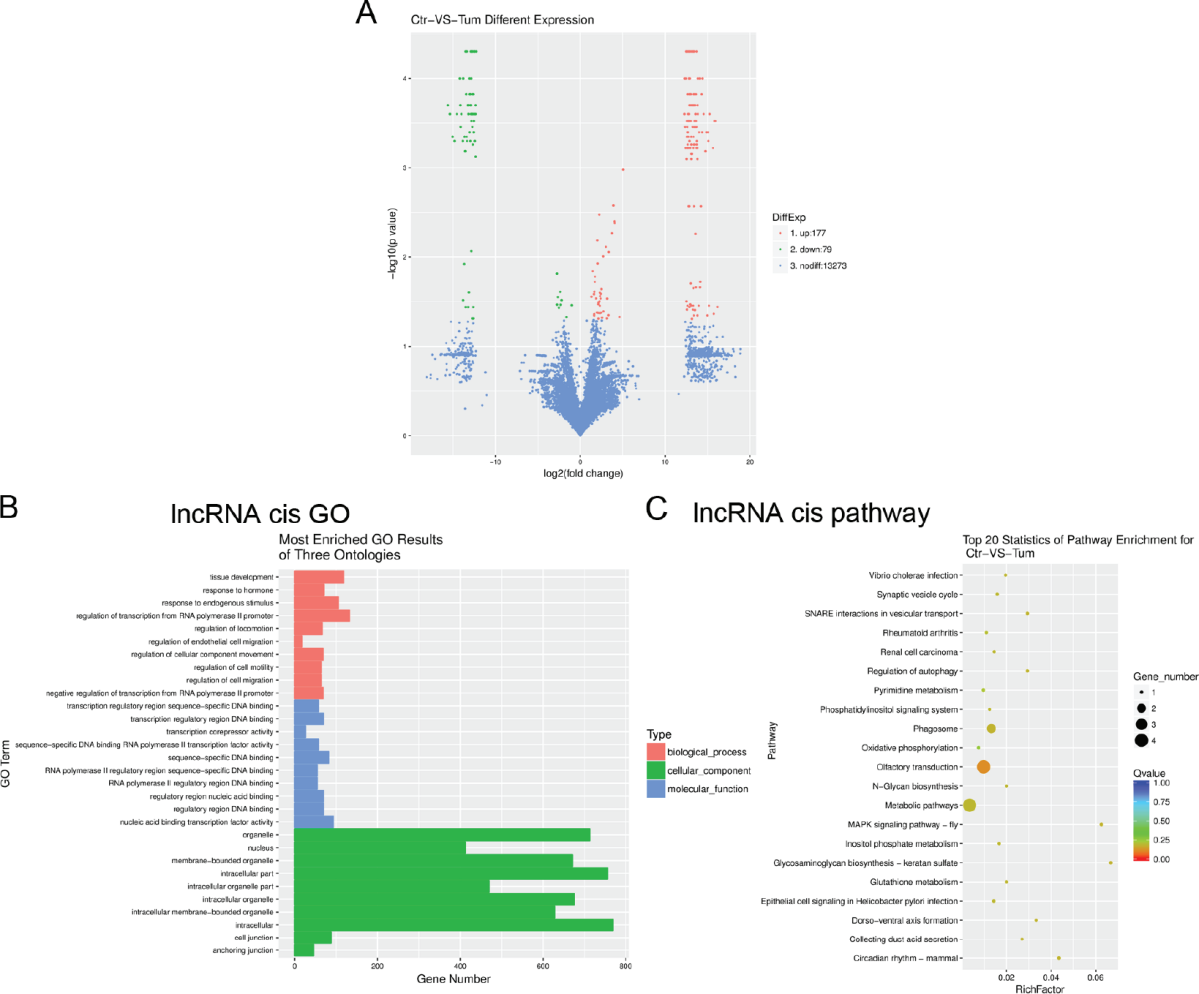
Gene ontology (GO) terms and pathways for target mRNAs of differentially expressed lncRNAs between infantile hemangioma and adjacent normal skin tissues (**A**) Hierarchical clustering shows a distinguishable lncRNA expression profiling among groups. (**B**) Most enriched GO terms of the three ontologies that are associated with the cis target mRNAs of differentially expressed lncRNAs are listed. (**C**) The top 20 pathways that are associated with the cis target mRNAs of differentially expressed lncRNAs are listed. (B) The horizontal axis represents the gene number. The term/GO on the vertical axis is drawn according to the first letter of the GO in descending order. Red bar represents the biological process, blue bar displays the molecular function, and green bar illustrates the cellular component. (C) The enrichment *Q* value or false discovery rate correct the *p* value for multiple comparisons. *P* values are calculated using Fisher’s exact test. The term/pathway on the vertical axis is drawn according to the first letter of the pathway in descending order. The horizontal axis represents the enrichment factor, i.e., (the number of dysregulated genes in a pathway/the total number of dysregulated gene)/(the number of genes in a pathway in the database/the total number of genes in the database). Top 20 enriched pathways are selected according to the enrichment factor value. The selection standards were the number of genes in a pathway ≥4. The different colours from green to red represent the *Q* value (False discovery rate value). The different sizes of the round shapes represent the gene count number in a pathway. Ctr, matched normal skin tissue; Tum, infantile hemangioma skin tissue.

Using the Gencode, RefSeq and UCSC Knowngene databases for non-coding transcripts, we identified 256 differentially expressed lncRNAs with greater than two-fold changes in IH and *p* values < 0.05 (Figure 2A). Of these, 177 were overexpressed and 79 were underexpressed in IH relative to the matched normal skin controls (fold change ≥ 2, *P* ≤ 0.05; for a list of differentially expressed lncRNAs, see Table 2). LncRNAs (long non-coding RNAs), are defined as greater than 200 nucleotides in length, transcribed by RNA polymerase II (RNA PII), and lacking an open reading frame [15]. LncRNAs have been found to regulate protein-coding (pc) gene expression at both the transcriptional and post-transcriptional levels [16]. To identify the potential mRNA targets of lncRNAs, we use RNAplex to predict the binding of lncRNAs with the antisense target mRNAs. mRNAs 10 kb upstream or downstream of lncRNAs were considered to be the conceivable lncRNA targets and defined as cis target mRNAs. Gene ontology (GO) analysis revealed that cis target mRNAs of differentially expressed lncRNAs were mostly involved in regulatory mechanisms related to transcription, nucleic acid binding transcription factor activity and intracellular components (Figure 2B). KEGG Pathway analysis indicated that the MAPK signalling pathway, regulation of autophagy and metabolic pathways were implicated for the cis target mRNAs of differentially expressed lncRNAs (Figure 2C).

**Table 2:** List of up-regulated and down-regulated lncRNAs detected using RNA-seq (FC ≥ 2.83, *P* < 0.05)

LncRNAID	GenePos	log2 (Tum/Ctr)	up-or-down	*p*_value	LncRNA GeneID
TCONS_00116532	chr5:101515283-101519050	–2.74762	down	0.0153	XLOC_074583
TCONS_00049881	chr16:76805140-76807266	–2.74208	down	0.03405	XLOC_031473
TCONS_00140157	chr8:111620546-111622506	–2.65655	down	0.02805	XLOC_090179
TCONS_00108080	chr4:97512593-97516549	–2.51518	down	0.03695	XLOC_068366
TCONS_00049878	chr16:76790731-76793194	–2.36369	down	0.0245	XLOC_031470
TCONS_00116531	chr5:101510927-101514253	–2.31673	down	0.03415	XLOC_074582
TCONS_00125444	chr6:77144877-77146646	–2.19865	down	0.0304	XLOC_080698
TCONS_00047386	chr15:95209116-95212765	–1.62607	down	0.04695	XLOC_030120
TCONS_00099093	chr3:117310248-117315662	1.60464	up	0.026	XLOC_061648
TCONS_00094390	chr3:115548706-115554914	1.63654	up	0.045	XLOC_058344
**TCONS_00112159**	**chr5:104342703-104346581**	**1.6409**	**up**	**0.0436**	**XLOC_071442**
TCONS_00099090	chr3:117295535-117304861	1.65949	up	0.0244	XLOC_061645
TCONS_00125869	chr6:92532111-92539501	1.72153	up	0.01895	XLOC_081015
TCONS_00092337	chr3:21220256-21226664	1.73612	up	0.01655	XLOC_056961
TCONS_00036940	chr13:105981280-105999840	1.8649	up	0.0291	XLOC_023137
TCONS_00022826	chr11:26799455-26802752	1.995	up	0.04175	XLOC_013761
TCONS_00092345	chr3:21256300-21262413	2.01955	up	0.0065	XLOC_056969
**TCONS_00125870**	**chr6:92539642-92542765**	**2.05275**	**up**	**0.0118**	**XLOC_081016**
TCONS_00144439	chr9:13841053-13843543	2.11565	up	0.04215	XLOC_093201
TCONS_00132673	chr7:94029530-94036098	2.1175	up	0.03365	XLOC_085154
**TCONS_00088818**	**chr22:11878998-11880540**	**2.12569**	**up**	**0.04895**	**XLOC_054900**
TCONS_00099084	chr3:117263272-117265249	2.13489	up	0.0322	XLOC_061639
TCONS_00089156	chr22:23536916-23548776	2.23141	up	0.00335	XLOC_055095
TCONS_00112938	chr5:136193267-136196387	2.2618	up	0.03135	XLOC_072057
TCONS_00093724	chr3:76746891-76748374	2.30541	up	0.02505	XLOC_057834
TCONS_00021543	chr11:124180846-124186471	2.31143	up	0.04245	XLOC_012894
TCONS_00099092	chr3:117307664-117309582	2.31645	up	0.0351	XLOC_061647
TCONS_00077659	chr2:12602918-12606149	2.33745	up	0.02735	XLOC_047338
TCONS_00106552	chr4:19193285-19198298	2.3654	up	0.02555	XLOC_067212
TCONS_00109906	chr4:187247968-187249825	2.40602	up	0.02915	XLOC_069754
TCONS_00106583	chr4:19283454-19288399	2.44245	up	0.0258	XLOC_067243
TCONS_00142894	chr8:98381305-98382530	2.46222	up	0.04195	XLOC_092151
TCONS_00092347	chr3:21263525-21265295	2.50214	up	0.0228	XLOC_056971
TCONS_00126048	chr6:104495681-104497908	2.57552	up	0.04765	XLOC_081156
TCONS_00021618	chr11:124429567-124433267	2.6993	up	0.00985	XLOC_012968
TCONS_00142579	chr8:89240840-89241898	2.72566	up	0.0405	XLOC_091914
TCONS_00116213	chr5:86348589-86351550	3.02011	up	0.0077	XLOC_074350
TCONS_00013577	chr10:64133769-64134877	3.14074	up	0.04865	XLOC_008097
TCONS_00021614	chr11:124419031-124423063	3.17524	up	0.02925	XLOC_012964
TCONS_00138013	chr8:9196728-9203476	3.31685	up	0.04465	XLOC_088644
TCONS_00117017	chr5:130223107-130225700	3.34741	up	0.0088	XLOC_074967
TCONS_00032295	chr12:91627173-91631603	3.74738	up	0.0054	XLOC_019826
TCONS_00116260	chr5:86539574-86547371	3.90234	up	0.00265	XLOC_074397
TCONS_00116261	chr5:86548066-86552126	4.02636	up	0.004	XLOC_074398
TCONS_00032240	chr12:91452883-91455961	4.05433	up	0.00415	XLOC_019771
TCONS_00087133	chr21:38178744-38181900	4.62793	up	0.04685	XLOC_053685
TCONS_00116241	chr5:86436761-86447930	5.05555	up	0.00105	XLOC_074378

Ctr, matched normal skin tissue; Tum, infantile hemangioma skin tissue.

### Differential expression and bioinformatics analysis of miRNAs in IH compared with matched normal skin controls

**Figure 3:**
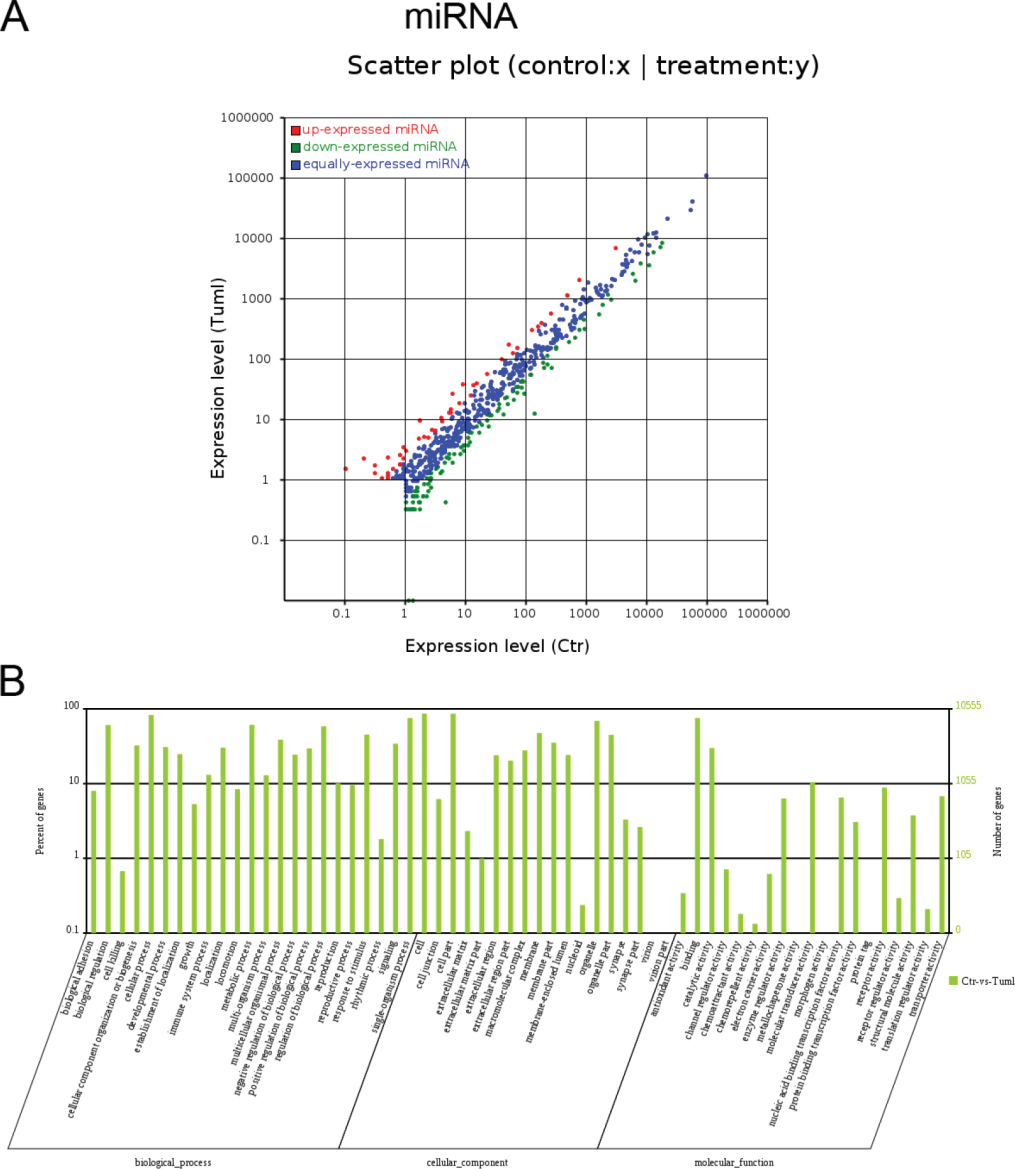
Expression profiles of differentially expressed miRNAs and Gene ontology (GO) terms for target mRNAs of differentially expressed miRNAs between infantile hemangioma and adjacent normal skin tissues (**A**) Scatter plot shows the differentially expressed miRNAs. Red dots represent up-regulated miRNAs. Green dots illustrate down-regulated miRNAs. (**B**) Most enriched GO terms of the three ontologies that are associated with the target mRNAs of differentially expressed miRNAs are listed. The term/GO on the horizontal axis is drawn according to the first letter of the GO in ascending order from left to right. The vertical axis represents the percent of genes and gene number. Ctr, matched normal skin tissue; Tum, infantile hemangioma skin tissue.

We also determines the miRNA expression profiles between IH and matched normal skin controls using HiSeq. One hundred forty-two miRNA candidates were found to be differentially expressed (fold change ≥ 2, *P* ≤ 0.05; for a list of differentially expressed miRNAs, see Table 3). Of these, 42 miRNAs were up-regulated in IH, whereas 100 miRNAs were down-regulated (Figure 3A). To examine the potential biological functions of the miRNAs of interest in IH, we use miRanda, targetscan and PITA software to identify the target genes of known miRNAs with differential expression profiles and extracted intersections or unions of target genes as the final prediction result. Gene ontology (GO) analysis revealed that the target mRNAs of differentially expressed miRNAs were mostly involved in cellular processes, cell components and binding (Figure 3B).

**Table 3:** List of up-regulated and down-regulated miRNAs detected using small RNA-seq (FC ≥ 2.83, *P* < 0.05)

miR_name	fold-change(log2 Tum/Ctr)	up-or-down	*p*_value	sig-lable
hsa-miR-9-3p	–7.10538475	down	0.000122742	**
hsa-miR-1303	–6.86430997	down	0.000490585	**
hsa-miR-223-3p	–3.53749571	down	1.65E-266	**
hsa-miR-509-3-5p	–3.49076386	down	4.54E-10	**
hsa-miR-509-5p	–2.50137905	down	0.00149862	**
hsa-miR-450a-2-3p	–2.22132264	down	0.007574552	**
hsa-miR-337-5p	–2.13638952	down	0.000918863	**
hsa-miR-135a-5p	–2.11442989	down	0.012782924	*
hsa-miR-513c-5p	–2.11442989	down	0.012782924	*
hsa-miR-2355-3p	–2.08634155	down	0.004368201	**
hsa-miR-202-5p	–1.99889374	down	0.007235532	**
hsa-miR-200c-5p	–1.99886536	down	0.021358055	*
hsa-miR-664b-3p	–1.99886536	down	0.021358055	*
hsa-miR-3648	–1.93753391	down	0.001441353	**
hsa-miR-429	–1.93708866	down	1.47E-256	**
hsa-miR-26a-1-3p	–1.92492452	down	0.004102228	**
hsa-miR-3611	–1.92492452	down	0.004102228	**
hsa-miR-187-3p	–1.89198508	down	0.000828053	**
**hsa-miR-664a-3p**	**–1.87801528**	**down**	**1.82E-41**	**
hsa-miR-383-5p	–1.87335512	down	0.03528718	*
hsa-miR-335-3p	–1.84083467	down	7.07E-87	**
hsa-miR-203a-3p	–1.72913403	down	0	**
hsa-miR-3912-3p	–1.71155782	down	2.01E-06	**
hsa-miR-183-3p	–1.69939239	down	0.031012226	*
hsa-miR-135b-5p	–1.69929888	down	3.86E-09	**
hsa-miR-141-5p	–1.69460943	down	6.85E-21	**
hsa-miR-377-5p	–1.65878433	down	7.03E-08	**
hsa-miR-16-5p	–1.65065115	down	0	**
hsa-miR-150-5p	–1.62494834	down	2.07E-49	**
hsa-miR-627-5p	–1.6233869	down	0.000309094	**
hsa-miR-6510-3p	–1.58388025	down	0.000271487	**
hsa-miR-548p	–1.58387326	down	0.026725738	*
hsa-miR-203b-3p	–1.58383438	down	1.74E-09	**
hsa-miR-195-5p	–1.5823761	down	0	**
hsa-miR-141-3p	–1.5772266	down	0	**
hsa-miR-200b-3p	–1.57584304	down	0	**
hsa-miR-944	–1.5589637	down	4.65E-10	**
hsa-miR-200b-5p	–1.53135387	down	2.72E-13	**
hsa-miR-31-5p	–1.5239109	down	6.16E-159	**
hsa-miR-183-5p	–1.52252398	down	2.78E-140	**
hsa-miR-92a-1-5p	–1.52249641	down	0.007046614	**
hsa-miR-493-5p	–1.51904529	down	6.20E-36	**
hsa-miR-320d	1.58604035	up	0.004519782	**
**hsa-miR-524-3p**	**1.69892552**	**up**	**4.81E-139**	**
hsa-miR-7704	1.8311765	up	0.000268926	**
hsa-miR-450a-1-3p	2.00090764	up	0.021183756	*
hsa-miR-185-5p	2.03429793	up	1.64E-39	**
**hsa-miR-503-5p**	**2.10316253**	**up**	**1.37E-29**	**
hsa-miR-122-5p	2.13865463	up	0.00090565	**
hsa-miR-1-3p	2.40555691	up	2.02E-13	**
hsa-miR-7-5p	2.40555691	up	2.02E-13	**
hsa-miR-520e	2.41594514	up	0.002562365	**
hsa-miR-1269b	3.39374391	up	3.56E-05	**
hsa-miR-1268a	3.8087166	up	0.000515933	**
hsa-miR-1268b	3.8087166	up	0.000515933	**

Ctr, matched normal skin tissue; Tum, infantile hemangioma skin tissue.

### Real-time quantitative PCR validation

**Figure 4:**
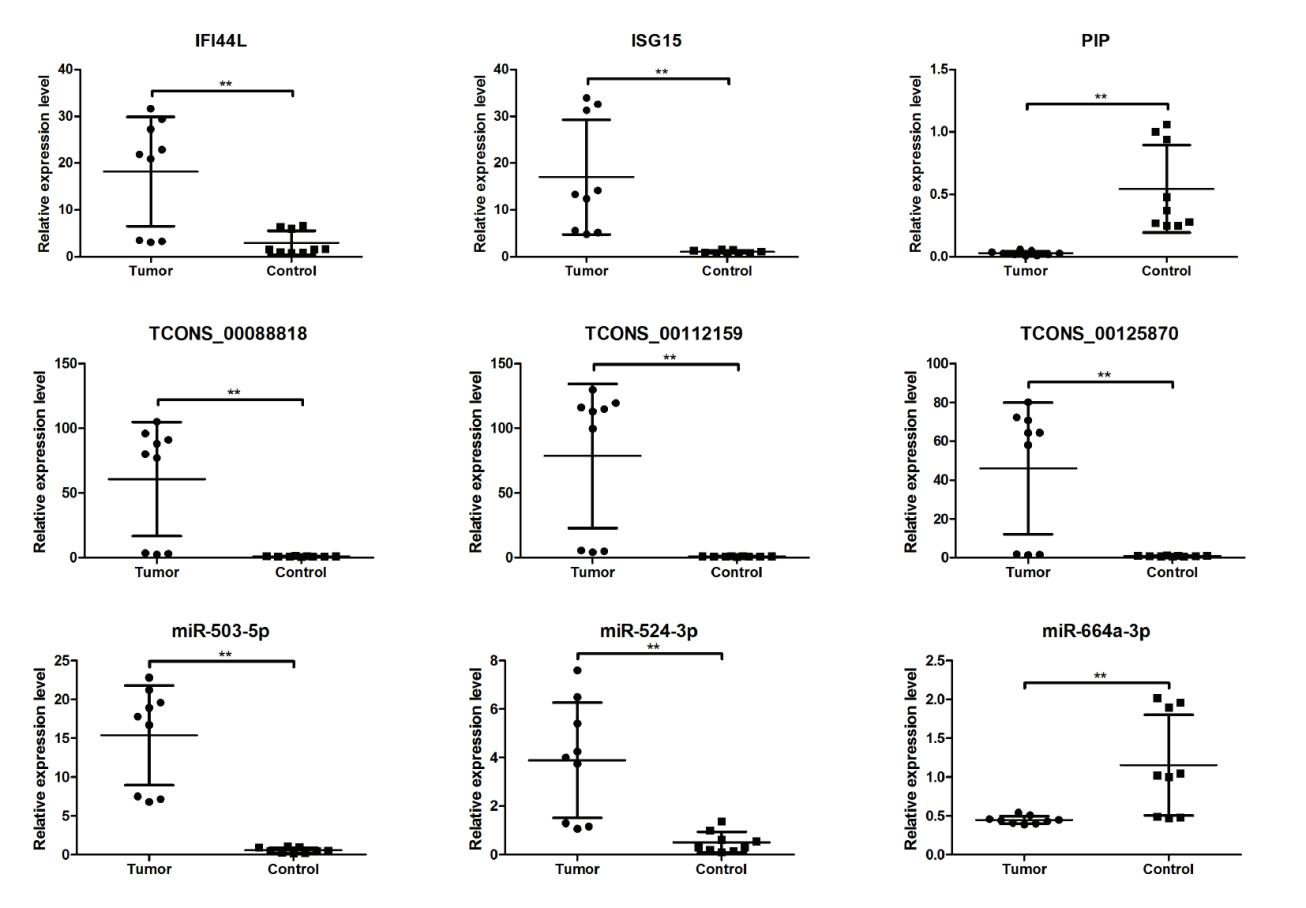
The differential expression of mRNAs, lncRNAs and miRNAs between additional IH skin (*n* = 9) and matched normal skin tissues (*n* = 9) mRNAs and lncRNAs expression were validated by quantitative real-time PCR using 2^(–△△Ct)^ method. miRNAs expression was validated by Bulge-Loop™ qRT-PCR. ^*^P < 0.05, ^**^P < 0.01.

To validate the RNA-seq data, we randomly selected 9 differentially expressed RNAs (bold-face type in Tables 1–3). Real-time quantitative PCR (qRT-PCR) and Bulge-Loop™ qRT-PCR analyses were performed on an additional 9 independent IH skin samples (Table 4). The results revealed that similar up-regulation or down-regulation patterns were observed in both the RNA-seq and qRT-PCR samples for the 9 RNAs (Figure 4, bold in Tables 1–3). Therefore, our RNA-seq data were reliable and stable. Among the 9 RNAs, IFI44L, ISG15, TCONS_00088818, TCONS_000112159, TCONS_000125870, miR-503-5p and miR-524-3p were all expressed to a greater extent in the IH tissues than in the matched normal skin controls.

**Table 4:** Demographic and clinical characteristics of infantile hemangioma (IH) patients (capillary hemangioma)

No.	Age	Gender	Position	Pathology	Sample use
1	3 months 8 days	Male	Left axilla	IH	RNA-seq
2	7 months 5 days	Female	Head	IH	RNA-seq
3	12 months	Female	Left Abdomen	IH	RNA-seq
4	9 months	Female	Right thoracic wall	IH	qRT-PCR
5	5 months	Male	Thoracic wall	IH	qRT-PCR
6	6 months 9 days	Female	Occiput	IH	qRT-PCR
7	8 months 13 days	Female	Right posterior neck	IH	qRT-PCR
8	10 months	Female	Left abdomen	IH	qRT-PCR
9	4 months	Female	Right thoracic wall	IH	qRT-PCR
10	11 months	Male	Thoracic wall	IH	qRT-PCR
11	8 months 21 days	Female	Occiput	IH	qRT-PCR
12	5 months 12 days	Female	Head	IH	qRT-PCR

### The ceRNA network construction

**Figure 5:**
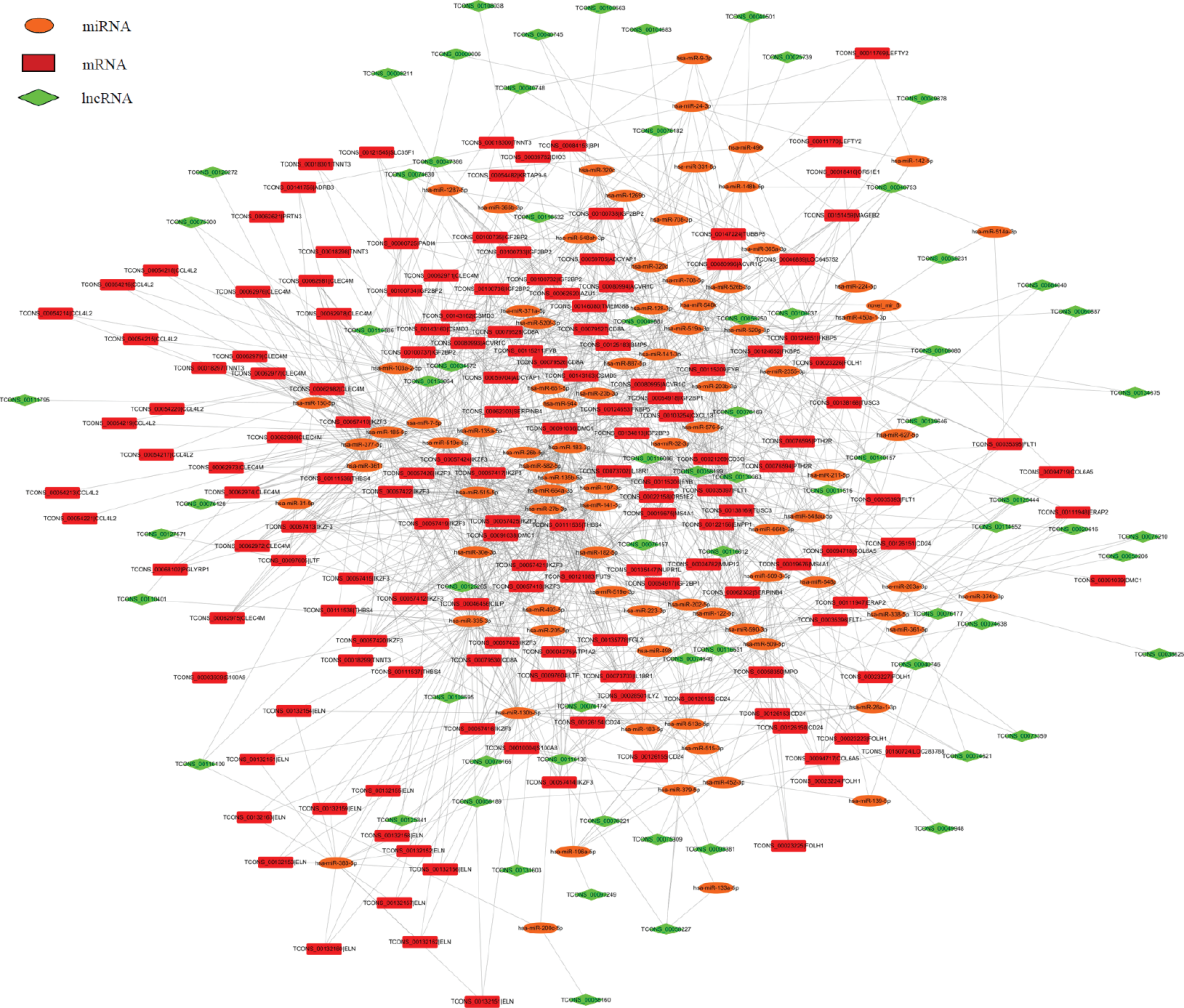
The ceRNA network of the differentially expressed miRNA-mediated lncRNAs and mRNAs interactions Red color represents the mRNA name, green color displays the lncRNA name, and orange color illustrates the miRNA name. Taken TCONS_00126150 | CD24 and TCONS_00126153 | CD24 as an example, the TCONS_ number before | means the ID number of each transcript, one gene has many transcripts.

Recently, lncRNAs and mRNAs have been demonstrated to be function as ceRNAs in diverse physiological and pathophysiological states. It is known that mRNAs or lncRNAs can bind miRNAs through microRNA response elements (MREs). Therefore, we use rna22 and targetscan to screen the lncRNAs and mRNAs with MREs. To ascertain the associations of differentially expressed lncRNAs and miRNAs with mRNAs, based on the 142 differentially expressed miRNAs, 144 differentially expressed mRNAs, and 256 differentially expressed lncRNAs, an lncRNA-miRNA-mRNA correlation network was constructed (Figure 5). The network displayed the associations among 87 miRNAs, 70 lncRNAs and 58 mRNAs mediated interactions. For example, miR-26a-1-3p could bind to lncRNAs TCONS_00074621 and mRNA CD24, miR-24-3p bind with TCONS_00000006 and LEFTY2, moreover, miR-514a-3p bind to TCONS_00040753 and FLT1. As shown in Figure 5, one miRNA was associated with one to tens of mRNAs and vice versa. One lncRNA was related to one to tens of miRNAs. In total, 1256 sponge modulators participated in 87 miRNA-mediated, 70 lncRNA-mediated and 58 mRNAs’ transcripts-mediated interactions. These findings indicated that the expression profiles of miRNAs, mRNAs and lncRNAs were significantly correlated.

## DISCUSSION

Currently, with the advent of next-generation sequencing technologies, RNA-Seq is gradually replacing microarrays for the detection of transcript expression profiling. IH is one of the most common tumors diagnosed in young children. The pathogenesis of hemangioma is largely unknown due to its sophisticated etiology. Although a lot of papers report the RNA networks in IH [12, 14, 17–19], those work all used microarrays methods. In this study, we used Ribo-Zero RNA-Seq and HiSeq means to examine the global expression profiles of protein-coding transcripts and non-coding RNAs, including miRNAs and lncRNAs, in IH and matched normal skin controls. Totally, 144 mRNAs, 256 lncRNAs and 142 miRNAs were found to be differentially expressed (fold change ≥ 2, *P* ≤ 0.05) in IH compared to matched normal skin. Further integrated ceRNA network analysis revealed that 353 sponge modulators participate in 39 miRNA, 29 lncRNA and 147 mRNA mediated interactions.

Competitive endogenous (ce) RNAs cross-regulate each other through sequestration of shared microRNAs and form complex regulatory networks based on their microRNA signatures [20]. Genome-wide transcriptional profiling of vessels from proliferating and involuting hemangiomas has been used to identify differentially expressed genes [19]. A lncRNA microarray study reported that a large number of genes are differentially expressed in IH [12]. Integrative meta-analysis identifies miRNA-regulated networks in IH [14]. Here, based on RNA-seq technology, using IH tissues and matched normal skin, we presented a new ceRNA network that determined the functions of particular miRNAs, lncRNAs and mRNAs in IH development (Figure 5). Alterations in one ceRNA may have striking effects on the integrated ceRNA and transcriptional networks. Taken miR-664a-3p as an example, it was down-regulated in IH tissues from the small RNA-seq data (Table 3). Bulge-Loop™ qRT-PCR demonstrated that expression of miR-664a-3p was decreased in another nine IH tissues (Figure 4). The ceRNA network analysis revealed that three lncRNAs TCONS_00020616, TCONS_00058199 and TCONS_00108595 could bind to miR-664a-3p. Moreover, nine mRNAs including ADCYAP1, CSMD3, IL18R1, IKZF3, CD8A, FGL2, FUT9, IGF2BP1, and TMEM38B were predicted to bind with miR-664a-3p (Figure 5). This ceRNA network indicated that those nine mRNAs’ expression could be regulated by miR-664a-3p, and three lncRNAs TCONS_00020616, TCONS_00058199, TCONS_00108595 could compete to bind with miR-664a-3p and then affect those nine mRNAs’ expression.

The pathogenesis of IH has been linked to pathways affecting angiogenesis and vasculogenesis [21]. Those microarray analyses concluded that angiogenin may be a useful serum marker for hemangiomas [22], IH endothelial cells (HEMECs) reflects a pro-proliferative cell type with altered adhesive characteristics [17], proliferating hemangiomas display increased expression of genes involved in endothelial-pericyte interactions, as well as those involved in neural and vascular patterning [19], lncRNAs likely regulate several genes in angiogenesis [12], miRNA-mRNA expression networks display that deregulated genes play roles in cell growth and differentiation, cell signaling, angiogenesis and vasculogenesis [14]. In the present study, using RNA-seq technology, we found that deregulated mRNAs related to immune system processes, carbohydrate derivative binding, extracellular region regulation, chemokine, NF-kappa B and TGF-beta signalling pathways, as well as cell adhesion molecules (CAMs) (Figure 1). Moreover, cis target mRNAs of differentially expressed lncRNAs were mostly involved in regulatory mechanisms related to transcription, nucleic acid binding transcription factor activity, intracellular components, MAPK signalling pathway, regulation of autophagy and metabolic pathways (Figure 2). Additionally, target mRNAs of differentially expressed miRNAs were mostly involved in cellular processes, cell components and binding (Figure 3). These results are partly consistent with those of previous studies in that CAMs are involved. Besides, RNA-seq data show some new findings in that regulation of autophagy and metabolic pathways, TGF-beta, NF-kappa B signalling and chemokine signalling were involved in the pathogenesis of IH.

Endothelial TGF-β signalling has been implicated in the regulation of angiogenesis [23]. Expression of NF-kappa B target genes was demonstrated in proliferating IH. Targeting NF-kappa B in infantile hemangioma-derived stem cells reduced VEGF-A expression [24]. The chemokine CXCL-14 has been reported to be involved in the occurrence and development of infantile hemangioma [25]. Our RNA-seq data found that TGF-beta, NF-kappa B signalling and chemokine signalling were involved in the pathogenesis of IH. The results are consistent with those of previous studies, which suggested that the RNA-seq data are reliable.

By carefully comparing our data with other’s, IGF2, FOXF1 and EGFL7 were reported to be up-regulated in IH, FOXC1 and EGFR were down-regulated in IH [12]. In this study, we found that IGF2 mRNA-binding proteins (IGF2BPs) including IGF2BP1, IGF2BP2 and IGF2BP3 were all downregualted in IH. Although recent publication reported that results obtained by RNA-seq and microarrays were highly reproducible [26], some discrepancy may be existed in the differentially expressed RNAs. Therefore, further demonstrating the function of particular RNA in IH development is urgently needed. In addition, larger samples are needed to perform receiver operating characteristic (ROC) curve analysis to prove that some of the IH RNAs are promising biomarkers.

Taken together, understanding the functional interactions among lncRNAs, miRNAs and mRNAs could lead to new explanations for IH disease pathogenesis [7]. Further elucidating the underlying mechanisms of the functions of miRNAs, lncRNAs and mRNAs in IH would be helpful in revealing the biological aetiology and potentially provide useful information for IH evaluation and treatment.

## MATERIALS AND METHODS

### Ethics statement

This study was approved by the Medical Ethics Committee of the Obstetrics and Gynaecology Hospital affiliated with Nanjing Medical University (No. [2015] 91). Children with IH underwent surgery at out hospital. The IH samples and matched normal skin controls were collected from patients who underwent surgery and whose parents provided written consent.

### Tissue samples

Proliferating capillary infantile hemangioma (IH) and matched normal skin tissues were obtained from 12 different patients who were admitted to the Obstetrics and Gynaecology Hospital affiliated with Nanjing Medical University for IH removal. Patient information is listed in Table 4. A diagnosis of proliferative infantile hemangioma was confirmed by routine pathological examination. The collected skin samples were immediately frozen in liquid nitrogen for total RNA preparation.

### Total RNA isolation

Total RNA was extracted from biopsy samples using the Qiagen RNeasy mini kit (Qiagen, Valencia, CA). After ribosomal RNA depletion, RNA-seq libraries were prepared using ScriptSeq complete kits from Epicenter (Madison, WI). RNA purity was assessed using the Nano Photometer^®^ spectrophotometer (IMPLEN, CA, USA), and RNA concentration was measured using the Qubit® RNA Assay Kit in Qubit^®^ 2.0 Flurometer (Life Technologies, CA, USA). RNA integrity was assessed using the RNA Nano 6000 Assay Kit from the Bioanalyser 2100 system (Agilent Technologies, CA, USA).

### Library preparation, quality examination and sequencing for mRNAs and lncRNAs

The sequencing libraries were prepared following manufacturer recommendations from the VAHTS™ Total RNA-seq (H/M/R) Library Prep Kit for Illumina^®^. The details of library construction were patented by the company (Vazyme, China). After cluster generation, the libraries were sequenced on an Illumina Hiseq X10 platform, and 150-bp paired-end reads were generated.

Raw reads in fastq format were first processed using in-house perl scripts. Clean reads were obtained by removing reads with adapters, reads in which unknown bases were more than 5% and low quality reads (if the percentage of low quality bases was greater than 50% in a given read, we defined the low quality base to be the base whose sequencing quality was no more than 10). At the same time, Q20, Q30, and GC contents were calculated for the clean reads. All downstream analyses were based on the clean reads.

The reference genome and gene model annotation files were downloaded directly from the genome website. The reference genome index was built using Bowtie (v2.1.0) [27], and the paired-end clean reads were aligned to the reference genome using TopHat (v2.1.1) [28].

### Transcriptome assembly, lncRNA prediction and target gene prediction

The mapped reads from each sample were assembled using Cufflinks (v2.2.1) [29] with a reference-based approach. Cufflinks uses a probabilistic model to simultaneously assemble and quantify the expression levels of a minimal set of isoforms, which provides a maximum likelihood explanation of the expression data in a given locus. Then, Cuffmerge was used to merge these sample assemblies into a master transcriptome, which was compared to known transcripts via Cuffcompare. The lncRNAs were predicted by several strict steps based on RNA structural characteristics and non-coding properties. The steps were as follows: 1) transcripts, not in any class code of “ j, i, o, u, x ”, were filtered out; 2) transcripts shorter than 200 bp were filtered out; 3) transcripts aligned to sequences in the NONCODE database [30] by blastn were identified as known lncRNAs; 4) the retained transcripts (known lncRNAs were not included) were used to predict protein coding potential by Coding Potential Calculator (CPC) [31] and TransDecoder (http://transdecoder.github.io/), transcripts with coding potential were removed, and those without coding potential were identified as novel lncRNAs. The known lncRNAs and novel lncRNAs were together used for subsequent analyses.

LncRNAs can negatively or positively affect expression of the downstream gene via an upstream noncoding promoter. Genes within 10 kb upstream or downstream of lncRNAs were abstracted by bedtools (http://bedtools.readthedocs.io/en/latest/) as lncRNA target genes. However, antisense lncRNAs can regulate overlapping sense transcripts. Transcripts that overlapped with LncRNAs on the opposite strand were also identified as lncRNA target genes, and the interactions between lncRNAs and transcripts were revealed by RNAplex [32].

### Quantification of gene expression levels and differential expression analysis

Cuffdiff (v2.2.1) [33] was used to calculate FPKMs for both lncRNAs and coding genes in each group. Gene FPKMs were computed by summing the FPKMs of the transcripts in each gene group. FPKM stands for “fragments per kilobase of exon per million fragments mapped” and is calculated based on the length of the fragments and the reads count mapped to each fragment.

Cuffdiff (v2.2.1) provides statistical routines for determining differential expression in digital transcripts or gene expression datasets using a model based on a negative binomial distribution. Transcripts or genes with corrected *p* values less than 0.05 and absolute values of log2 (fold change) <1 were classified as significantly differentially expressed.

### Small RNA sequencing and bioinformatics analysis

Total RNA was separated by 15% agarose gels to extract the small RNA (18–30 nt). After ethanol precipitation and centrifugal enrichment of small RNA samples, the library was prepared according to the methods and processes described in the Small RNA Sample Preparation Kit (Illumina, RS-200-0048). Insert size was assessed using the Agilent Bioanalyser 2100 system (Agilent Technologies, CA, USA), and after the insert size was consistent with expectations, qualified insert size was accurately quantitated using a Taqman fluorescence probe from the AB Step One Plus Real-Time PCR system (Library valid concentration > 2 nM). The qualified libraries were sequenced using an Illumina Hiseq 2500 platform, and 50-bp single-end reads were generated.

First, the tags were mapped to the reference genome by SOAP [34] to analyse their distributions within the genome and were aligned to the miRBase database [35] using blast. The tags were identified as known miRNAs when they satisfied the following criteria: 1) there were no mismatches when aligned to the miRNA precursors in the miRBase database; 2) based on the first criteria, the tags were aligned to the mature miRNAs in the miRBase database with at least 16-nt overlap while allowing offsets. The miRNA target genes were predicted using two software programs (targetscan and miRanda) as we previously described [36], and the intersection of target genes (the intersections were the same target genes of the same miRNAs) were the final target genes.

The miRNA expression levels were measured by “Transcripts Per Kilobase Million” (TPM).

$$\mathrm{TPM}=10^{6}C/L$$

where C is the read count of a miRNA and L is the total count of clean reads in sample.

Differentially expressed miRNAs were evaluated using the following statistical tests:

1) Statistical algorithm developed by Audic and Claverie (1997) [37]

$$P\left(y|x\right)={\left[\frac{N_{2}}{N_{1}}\right]}^{y}\frac{\left(x+y\right)!}{x!y!{\left[1+\frac{N_{2}}{N_{1}}\right]}^{\left(x+y+1\right)}}$$

where N_1_ is the total clean reads from sample 1, N_2_ is the total clean reads from sample 2, x is the number of reads from sample 1 mapped to miRNA A and y is the number of reads from sample 2 mapped to miRNA A.

### Gene Ontology (GO) and KEGG enrichment analysis

GO enrichment analysis of differentially expressed genes or target genes of differentially expressed lncRNAs was implemented using a perl module (GO::TermFinder) [38]. GO terms with corrected *p* values less than 0.05 were considered to be significantly enriched among the differentially expressed genes or the target genes of differentially expressed lncRNAs. R functions (phyper and qvalue) were used to test for statistical enrichment of the differentially expressed genes or target genes of the differentially expressed lncRNAs among the KEGG pathways. KEGG pathways with corrected *p* values less than 0.05 were considered to be significantly enriched among the differentially expressed genes or the target genes of the differentially expressed lncRNAs.

### Validation of RNA-seq data

To confirm the RNA-seq data, the expression profiles of randomly selected mRNAs and lncRNAs were tested in another 9 IH patients using quantitative real-time polymerase chain reactions (qRT-PCR) with the SYBR green method on an Applied Biosystems ViiA™ 7 Dx (Life Technologies, USA). Patient information is listed in Table 4. The sequences of the specific PCR primer sets used for qRT-PCR are listed in Table 5. The RNA expression levels were normalized to the internal control gene, glyceraldehyde 3-phosphate dehydrogenase (GAPDH), using the 2^(–△△Ct)^ method as we previously described [39]. Three selected miRNAs were further examined by Bulge-Loop™ qRT-PCR according to the manufacturer’s protocol (RIBOBIO, Guangzhou, China) with the SYBR green method on an Applied Biosystems ViiATM 7 Dx (Life Technologies, USA). The miRNA expression levels were normalized to u6 (RIBOBIO, Guangzhou, China), using the 2^(–△△Ct)^ method.

**Table 5:** Details of primer pairs used in analysis of mRNAs and lncRNAs expression by qRT-PCR

Gene name	Forward primer (5′-3′)	Reverse primer (5′-3′)
IFI44L	ACAGAGCCAAATGATTCCCTATG	TCGATAAACGACACACCAGTTG
ISG15	CGCAGATCACCCAGAAGATCG	TTCGTCGCATTTGTCCACCA
PIP	GTCAGTACGTCCAAATGACGAA	CTGTTGGTGTAAAAGTCCCAGT
TCONS_00088818	GCCTTGTGGTGTCTCCTCAG	TAGACCAGGCGTCATAGCAGAA
TCONS_00112159	GAAACAGCCACGGAGGGAAC	GATTTCTGCAATGCCGTGCC
TCONS_00125870	CCTAGAACCAGGGGCCACAA	TTTGCTGGGCACTCTGTAGC

### CeRNA network analysis

The miRanda and TargetScan assessments were used to identify ceRNAs (competing endogenous RNAs, including protein-coding messenger RNAs, long non-coding RNAs and circular RNAs), containing microRNA response elements (MREs). Then, ceRNAs with common miRNAs were selected to predict the global interactions between miRNAs and ceRNAs. Additionally, the ceRNAs with common miRNAs that were up- or down-regulated by miRNAs were abstracted based on differential expression to predict the co-regulated interactions of miRNAs and ceRNAs. The co-regulated ceRNA network was generated by Cytoscape (V. 3.4.0) [40].

### Statistical analysis

Data were analysed using the SPSS 20.0 software package (SPSS, Chicago, IL, USA) with an independent-samples *T* test performed between the two groups. All values are represented as the mean ± standard deviation (SD) from at least three independent experiments. Statistical significance was defined as *P* < 0.05.
